# Validation of Polish version of the Basel Extent of Rationing of Nursing Care revised questionnaire

**DOI:** 10.1371/journal.pone.0212918

**Published:** 2019-03-20

**Authors:** Izabella Uchmanowicz, Marcia Kirwan, Olga Riklikiene, Renata Wolfshaut–Wolak, Joanna Gotlib, Maria Schubert

**Affiliations:** 1 Department of Clinical Nursing, Faculty of Health Sciences, Wroclaw Medical University, Wroclaw, Poland; 2 Department of Nursing and Human Sciences, School of Nursing and Human Sciences, Dublin City University, Dublin, Ireland; 3 Department of Nursing and Care, Faculty of Nursing, Lithuanian University of Health Sciences, Kaunas, Lithuania; 4 Department of Epidemiology and Population Studies, Faculty of Health Sciences, Jagiellonian University Medical College, Cracow, Poland; 5 Division of Teaching and Outcomes of Education, Faculty of Health Sciences, Medical University of Warsaw, Warsaw, Poland; 6 Institute of Nursing, School of Health Professions, ZHAW Zurcher University of Applied Science, Winterthur, Switzerland; Nord University, NORWAY

## Abstract

Development of simple, valid and reliable instruments to determine nursing care rationing is a subject of ongoing research. One such instrument, which is gaining popularity worldwide and has significant research applicability, is the Basel Extent of Rationing of Nursing Care (BERNCA) and its revised version, the BERNCA-R. The aim of this study was to translate and adapt the BERNCA-R into a Polish-language version and to assess its reliability and validity in evaluating the level of implicit rationing of nursing care in Poland. Standard methodological requirements were followed during translation and cultural adaptation of the English version of the BERNCA-R questionnaire into Polish. The cross-sectional validation study was conducted between May and September 2017, which included 175 nurses undergoing specialisation and qualification courses at the European Postgraduate Education Centre in Wrocław, Poland. Cronbach’s alpha and inter-item correlations were used to analyse the internal consistency of the Polish BERNCA-R questionnaire. The mean total BERNCA-R score was 1.9 points (SD = 0.74) on a scale of 0–4. Cronbach’s alpha for the unidimensional scale was 0.96. The mean inter-item correlation was 0.4 (range 0.1–0.84), which indicates high internal consistency. A single-factor solution demonstrated stable loadings above 0.5 for almost all items of the Polish BERNCA-R questionnaire. The study using the Polish BERNCA-R questionnaire demonstrated that the instrument is valid and reliable for use in investigating care rationing in groups of Polish nurses.

## Introduction

### The phenomenon of implicit rationing of nursing care

Implicit rationing of nursing care occurs when the available resources are not sufficient to provide the necessary care to all patients. Implicit implies that no rules and regulations are available for guiding these rationing processes. Therefore, the individual nurse makes the decision as to decide how scarce resources are distributed among the patients and who will or will not receive an element of necessary nursing care [1–5]. It has been shown in the most recent systematic review that missed care is a promising indicator of nurse staffing adequacy, however, it should be noted that so far there is still little evidence that adding support workers to the team reduced missed care [6].

The reasons for this situation include staff reductions, the increased demand for care due to technological advancements, more treatment options and more informed service users, all of which require more time and attention from care professionals. Rationing of nursing care occurs during the process of care at the nurse-to-patient interface. The restriction can be seen as an end product of clinical judgment and decision-making processes when resources are not sufficient to provide all necessary nursing care to all patients. In this case, the responsible nurse has no other option than to ration certain aspects of necessary care [1].

The evidence indicates that the rationing of nursing care is largely affected by the decisions that nurses make, using their clinical judgment and knowledge about how to allocate already scarce resources, along with the broader values related to care in our society [5,7]. Additionally, the quality of the nursing practice environment (i.e. adequate staffing and resources, teamwork, leadership and autonomy) and patient safety have an impact on levels of rationed care [8].

### Nurse workforce in Poland

Current projections indicate that the number of practicing nurses in Poland is expected to decrease and that by 2020 the number of registered nurses will diminish by 23,000. An analysis of the ratio of the number of registered nurses to the number of inhabitants revealed that in 2016 there were 6.25 nurses per 1000 of the population and data suggest that the projected ratio will continue to decrease, with a value of 5.67 in 2020, 4.87 in 2025 and 4.01 in 2030 [9].

According to simulations carried out by the Central Register of Nurses and Midwives, a decline in the ratio of the number of registered nurses per 1000 of the population will be seen in 2030 in all Polish provinces. The expected ratio of the number of practicing nurses per 1000 of the population during 2016–2030 will be the lowest in the Lower Silesian and Silesian provinces, a decrease of 2.44 and 2.42, respectively [9]. Similar forecasts for 2030 were presented in 2016 by the Polish National Health Fund that projects that the nursing employment rate will continue to decline in all provinces [10].

Immigration has a significant influence upon maintaining a sustainable workforce in the field of healthcare in many countries [11]. According to the World Health Organization (WHO) [12], there will be a shortage of 12.9 million healthcare workers worldwide in 2035. The growth of these health professionals, including nurses, is driven mainly by an increase in demand for medical and care services in all European countries and a legal framework that allows for mutual recognition of professional qualifications acquired in European Union (EU) Member States [13,14].

An analysis by the European Commission [15] demonstrated that Polish nurses were ranked in the fifth position in terms of the number of emigrants (after Romania, Spain, Portugal, and Germany). The exodus of healthcare professionals, doctors and nurses included, is particularly evident from Eastern and Southern European countries to Western and Northern ones. The largest beneficiaries of this movement are the United Kingdom, Ireland, Austria, Sweden, Denmark and Belgium.

The projected decline in the Polish nurse workforce is likely to have an enormous impact on care provision to patients in Poland. Evidence from other countries (REF) would imply that as the workforce reduces, more practising nurses in the Polish health system will find themselves making decisions around care rationing in their daily work lives. For this reason it is important to ensure that a valid and reliable instrument exists to help measure and address this phenomenon in Poland.

### Measurement instrument for rationing of nursing care

Implicit rationing of nursing care was studied originally by Schubert et al. [1,4] in the context of the Rationing of Nursing Care in Switzerland (RICH) nursing study. In order to measure rationing of nursing care in that country, the researchers developed the Basel Extent of Rationing of Nursing Care (BERNCA) instrument.

The results of the RICH nursing study indicate that rationing of nursing care is a relevant system factor, which is linked to patient outcomes. Furthermore, it is clear that even low levels of rationing have a negative effect on patient outcomes. The results of this and other studies indicate that higher levels of rationing are linked with negative patient outcomes, poor quality of care and adverse incidents (such as medication errors), pressure ulcers and even increased mortality [1,16–19].

Rationing of nursing care, which is caused primarily by staff shortages, is becoming an increasingly serious issue. Insufficient staffing has serious consequences since certain tasks related to patient care cannot be performed. Identifying levels of care rationing, which may have a number of adverse consequences for patients, is particularly important [20]. Such evaluation is not, however, possible without an instrument in Polish that will help estimate the extent of care rationing in a group of Polish nurses.

We aimed to translate and adapt the BERNCA-R into a Polish-language version and to evaluate the reliability and validity in evaluating the level of implicit rationing of nursing care in Poland.

## Material and methods

### Evidence based on test content

The content testing was evaluated using two steps: (1) verification of the understandability, clarity and acceptability of the translated version and (2) pilot testing to evaluate the content validity.

#### First step

The translations were evaluated by a panel of experts from the Jan Mikulicz-Radecki Memorial University Clinical Hospital in Wroclaw, Poland, comprised of 10 nurses with Master’s degrees in Nursing and a minimum of five years of work experience in the field. The panel verified the phrasing and the meaning of all questions, as well as the clarity and accuracy of the instructions. The version selected by the panel subsequently underwent back-translation and the result was submitted for equivalency checking by the author of an English version.

#### Second step

Following this approval, the preliminary version was piloted in a group of 30 nurses undergoing specialist training at the Internal Nursing Care of the European Postgraduate Education Centre in Wrocław, Poland. Eventually, the final Polish version of the BERNCA-R questionnaire was obtained and analysed according to the study design.

### BERNCA-R questionnaire

The BERNCA-R questionnaire included 32 items, detailing necessary nursing tasks including daily activities, emotional or psychosocial support, education and rehabilitation care, safety conditions, and documentation. Using a 5-point Likert-type scale [0 = not required, 1 = never, 2 = rarely, 3 = sometimes, 4 = often), the respondents were asked to rate how frequently in the past seven working days they were unable to perform the 32 tasks due to inadequate time, staffing levels and/or skill mix. Results indicated that when patients reported higher levels of nursing care rationing, they were less likely to recommend the hospital to their family member or friends. The initial validity (content and construct validity) and reliability of the BERNCA questionnaire were established using nurse survey data from five German-speaking Swiss hospitals [4].

An explanatory factor analysis confirmed the internal structure and the hypothesised unidimensionality of the scale (construct validity); Cronbach’s alpha was 0.93 [4]. To calculate the average level of implicit rationing of nursing care, the scores for each nurse were averaged over all 32 items; the summary score ranged from 0 to 60, while the means ranged from 0 to 3.0 [4]. Schubert et al. revised the BERNCA for the use in the Registered Nurse Forecasting (RN4CAST) study including a sample of 35 acute care hospitals from the German, French and Italian speaking regions of Switzerland.

The revised BERNCA-R includes 32 items with necessary nursing tasks. In the context of the revision, seven items with double content have been reformulated into items with single content and five items with medical, technical and therapeutic treatment measures have been added. In order to differentiate between unnecessary tasks and never-rationed tasks, the rating scale has been altered from a 4-point to a 5-point Likert scale (0 = not required, 1 = never, 2 = rarely, 3 = sometimes, 4 = often) [17]. In this study, we used the revised BERNCA-R version with 32 items.

### Translation and adoption

Cross-cultural adaptation of the original English version of the BERNCA-R for Poland was performed in accordance with the Brislin method of using standard back-translation. This method is strongly recommended for cross-cultural research [21]. The English version of the BERNCA-R was forward-backward translated by native, bilingual Polish and English speakers to ensure equivalence across languages and cultures.

Having received the authors’ approval, the English version of the questionnaire was translated into Polish by an independent native Polish speaker. An independent native (bilingual) speaker then translated the Polish version back into English. Translators were professionals and fluent in English professional language and terminology. Members of the research team compared the backward translation with an English-language version of the BERNCA-R. The difference in the translation was discussed with translators and the developer of the original questionnaire and corrected as required.

### Validity and reliability testing

The validity of the Polish BERNCA-R version was evaluated in view of the evidence based on content and internal structure (content and construct validity). The dependability of the translated version was evaluated using internal consistency and inter-rater reliability.

### Setting and participants

A cross-sectional study was conducted between May and September 2017 at the European Postgraduate Education Centre in Wrocław, Poland. The study included only registered nurses with a Polish nursing diploma (BNS: bachelor's or MNS: master's degree in nursing sciences) and a minimum of two years of work experience in direct patient care. Lack of consent to participate in the study and the fact of being a student nurse were the only criteria for exclusion.

A total of 175 nurses undergoing specialisation and qualification courses at the European Postgraduate Education Centre in Wrocław, Poland were finally included to participate in the study. Data collection was performed consecutively within a period of 7 months. All nurses who met the inclusion criteria were invited to fill out the BERNCA-R questionnaires. The questionnaires were distributed by members of the research team during the course and were collected in a closed box placed on the classrooms.

### Study ethics

All respondents consented to participate in writing and the study protocol was approved by the Independent Bioethics Committee of the Wrocław Medical University (decision no. 282/2017 of May 4, 2017).

### Statistical analysis

The development of the Polish version and the testing of the translated BERNCA-R was done by examining responses, internal structure and reliability. Cronbach’s alpha and inter-item correlations were used to calculate the reliability of the instrument’s overall scale and extracted subscales, as well as the Guttman split-half reliability coefficient. A Cronbach alpha of greater than or equal to 0.6 was interpreted as acceptable [22]. The analysis of each quantitative variable for a total BERNCA-R score was conducted by calculating the mean (M), standard deviation (SD), median (Me), lower (Q1) and upper (Q3) quartiles, and minimum (Min) and maximum (Max) values. Quantitative variables (nominal and ordinal) were presented detailing the response number (n) and the percentage (%). The sample size was based on literature data, indicating that the minimum number of participants in a validation study should be five times the number of variables analysed [23]; therefore, a validation study for the BERNCA-R should include a minimum of 100 nurses (5x32 items). Statistical data analyses were performed using R package, version 3.4.2.

## Results

The study included 175 nurses and detailed characteristics of the participants are shown in Table 1.

**Table 1:** Respondents’ characteristics.

Characteristic (n = 175)		n	%
Age	20–30 years	23	13.14%
	31–40 years	16	9.14%
	41–50 years	89	50.86%
	51–60 years	44	25.14%
	61 years or more	3	1.71%
Sex	Female	169	96.57%
	Male	5	2.86%
	No data	1	0.57%
Education	Medical high school	63	36.00%
	Post-secondary medical course	28	16.00%
	Bachelor’s degree in nursing	46	26.29%
	Master’s degree in nursing	33	18.86%
	Other	4	2.29%
	No data	1	0.57%
Work experience as a nurse	0–5 years	26	14.86%
	6–10 years	16	9.14%
	11–15 years	9	5.14%
	16–20 years	19	10.86%
	21 years or more	103	58.86%
	No data	2	1.14%
Postgraduate education*	Specialist courses	111	63.43%
	Qualification courses	80	45.71%
	Specialization	38	21.71%
	Other	26	14.86%

* The total exceeds 100%, as the item allowed for multiple choices.

A principal component analysis was conducted to evaluate the internal structure of the Polish BERNCA-R. In the principal component analysis, as many as eight factors with eigenvalues exceeding 1 were found, and another two having values nearing 1. The first one accounted for 45.2% of the variance, while the remaining one accounted for less than 10%. Therefore, a single-factor solution was selected, which demonstrated stable loadings above 0.5 for 31 of 32 items; the loading for item 20 was very close to 0.5, as seen in Table 2.

**Table 2:** Factor loadings from exploratory factor analysis.

Item	Factor loading	Item	Factor loading	Item	Factor loading	Item	Factor loading
1	0.58	9	0.72	17	0.68	25	0.67
2	0.65	10	0.71	18	0.64	26	0.58
3	0.7	11	0.67	19	0.7	27	0.53
4	0.67	12	0.68	20	0.49	28	0.60
5	0.7	13	0.79	21	0.53	29	0.64
6	0.68	14	0.68	22	0.53	30	0.71
7	0.64	15	0.76	23	0.68	31	0.73
8	0.61	16	0.58	24	0.71	32	0.72

Kaiser-Meyer-Olkin (KMO) test scores at 0.9 and the statistically significant result of the Bartlett test (χ² = 3415.559; p<0.001) indicate that factor analysis is warranted, as the item correlation matrix is factorable.

### Validity

#### Missing data

Percentages of missing data were small, between 0.00% (items 1, 2, 3) and 7.43% (item 28). The missing data percentage exceeded 5% for items 12, 19, 20 and 25. The overall missing data percentage was 2.84%. Of the 130 respondents (74.29%) who completed the entire questionnaire, 23 (13.14%) missed 1 item, 11 (6.29%) missed 2 items and 11 (6.29%) missed 3 or more items.

#### Total score distribution

The mean total BERNCA-R score was 1.9 points (SD = 0.74) on a scale of 0–4. The median score was 1.91 points. The first and third quartiles were 1.37 and 2.33 points, respectively. Typical scores in the groups ranged between 1.37 and 2.33 points, as seen in Table 3 and in S1 Fig.

**Table 3:** Descriptive statistics of the total BERNCA-R score.

Total BERNCA-R score							
N	Mean	SD	Median	Min.	Max.	Q1	Q3
175	1.9	0.74	1.91	0	3.81	1.37	2.33

#### Item score distribution

No significant floor or ceiling effects were found. The means for individual items ranged between 1.49 (SD = 1.12, item 1) and 2.35 (SD = 1.16, item 29), as seen in Table 4.

**Table 4:** Descriptive statistics of BERNCA-R item responses (N = 175).

Item	There was no need [%]	Never [%]	Rarely [%]	Sometimes [%]	Often [%]	No data [%]	Mean	SD
1	17.71	41.71	21.71	12.00	6.86	0.00	1.49	1.12
2	14.86	40.57	28.57	12.00	4.00	0.00	1.5	1.02
3	14.86	32.00	27.43	17.14	8.57	0.00	1.73	1.17
4	13.14	24.57	26.86	21.14	11.43	2.86	1.93	1.22
5	12.57	24.57	28.00	18.86	12.57	3.43	1.94	1.22
6	9.71	40.00	32.00	13.14	3.43	1.71	1.6	0.96
7	9.71	21.14	28.57	21.71	14.29	4.57	2.1	1.21
8	10.29	24.00	30.29	24.57	10.29	0.57	2.01	1.15
9	10.86	29.71	32.00	13.71	12.00	1.71	1.86	1.17
10	9.14	22.86	31.43	21.14	14.86	0.57	2.1	1.19
11	6.29	17.71	38.86	18.86	17.71	0.57	2.24	1.13
12	5.71	24.57	33.14	18.86	12.00	5.71	2.07	1.1
13	14.29	22.29	28.00	21.71	13.14	0.57	1.97	1.25
14	14.86	28.57	26.86	18.86	10.29	0.57	1.81	1.21
15	18.29	9.71	30.29	21.14	19.43	1.14	2.14	1.35
16	18.29	23.43	27.43	17.71	10.29	2.86	1.78	1.25
17	7.43	26.86	29.71	19.43	13.14	3.43	2.04	1.16
18	2.86	30.29	31.43	22.29	10.29	2.86	2.07	1.04
19	3.43	21.71	36.57	20.57	12.00	5.71	2.17	1.04
20	20.57	19.43	20.57	23.43	10.86	5.14	1.84	1.33
21	21.71	25.14	16.57	22.86	10.86	2.86	1.75	1.34
22	10.86	30.86	28.57	18.86	6.86	4.00	1.79	1.1
23	6.29	21.14	33.71	26.86	9.14	2.86	2.12	1.06
24	9.14	34.29	30.29	18.29	5.14	2.86	1.75	1.04
25	5.71	45.14	29.14	10.29	3.43	6.29	1.58	0.9
26	9.14	29.71	27.43	22.29	8.57	2.86	1.91	1.12
27	4.00	55.43	22.29	9.71	5.14	3.43	1.55	0.93
28	4.57	48.00	26.29	9.71	4.00	7.43	1.57	0.9
29	5.14	19.43	26.86	26.86	18.29	3.43	2.35	1.16
30	5.71	21.14	29.71	28.00	11.43	4.00	2.19	1.09
31	7.43	20.00	32.00	26.86	9.71	4.00	2.12	1.09
32	4.00	24.57	35.43	21.71	11.43	2.86	2.12	1.05

### Reliability

Cronbach’s alpha for the scale was 0.959; the mean inter-item correlation was 0.4 (range 0.1–0.84), which indicates high scale consistency. The Guttman split-half reliability coefficient was 0.974, which indicates a perfect reliability. The average value of the item-scale correlation coefficient between the obtained scale was 0.62 (range 0.5–0.76), which indicates high scale consistency, as in Table 5.

**Table 5:** Inter-scale correlations for BERNCA-R items.

Item	Item-scale correlation	Item	Item-scale correlation	Item	Item-scale correlation	Item	Item-scale correlation
1	0.54	9	0.7	17	0.65	25	0.62
2	0.57	10	0.69	18	0.63	26	0.57
3	0.61	11	0.64	19	0.67	27	0.5
4	0.62	12	0.66	20	0.5	28	0.53
5	0.68	13	0.76	21	0.55	29	0.58
6	0.6	14	0.66	22	0.52	30	0.65
7	0.6	15	0.72	23	0.66	31	0.68
8	0.6	16	0.55	24	0.67	32	0.68

## Discussion

The purpose of this study was to prepare a Polish adaptation of the BERNCA-R questionnaire and to validate the instrument with a group of Polish nurses. The original BERNCA was developed based on the previously described conceptual framework of implicit rationing of nursing care, as well as on preliminary evidence, the clinical expertise of members of the research team and the Swiss Red Cross (Schweizerisches Rotes Kreuz; SRK) framework for nursing education (SRK, 1992). The original BERNCA version was validated within the RICH nursing study [4].

Many studies have provided evidence that nurse understaffing and an unsupportive work environment may lead to adverse patient outcomes, such as increased morbidity and mortality, in addition to higher costs [19,24,25].

Rationed omitted nursing care can be seen as an indicator of the quality and processes of nursing care being delivered across settings [8,26]. As previously discussed, low nurse staffing levels are associated with the increased risk of negative outcomes in acute care hospitals. There is a lack of knowledge about the decision-making process or criteria used by nurses when distributing scarce resources among their patients, and about the factors that may influence the implicit rationing of care [2,4,27]. Such knowledge would enable Health Services to look at interventions or strategies to use when the measured rationing levels exceed the defined thresholds [28].

The Polish validation was based on determining a routine psychometric characteristic–Cronbach's alpha, measuring the instrument's internal consistency. The internal consistency of the Polish BERNCA-R questionnaire was 0.96.

According to most authors, the optimum internal consistency is indicated by Cronbach’s alpha at ≥ 0.90; values ≥ 0.80 are considered good, ≥ 0.70 are acceptable, ≥ 0.60 are questionable, ≥ 0.50 are poor and < 0.50 are unacceptable [29]. In this context, the Polish version of the BERNCA-R questionnaire should be considered to be a highly valuable instrument.

We have used BERNCA-R for validation because data suggests that rationing scores within BERNCA can provide a clinically meaningful method for tracking the effects of low resources or other difficulties in allocating resources to patient outcomes [28]. Studies that measure the rationing of nursing care that arises from a shortage of nurses are extremely valuable. Some evidence suggests that care left undone is strongly related to the nurses’ overall perceptions of the quality and safety of care [30–32].

The BERNCA hospital version was first available in German, French and Italian from the Swiss branch of the RN4CAST Study [17]. It should be noted that although other countries have used the BERNCA, to the best of our knowledge these versions have not been published. Based on this study, there will also be a Polish version of this measurement tool. It is important to emphasise that the BERNCA-R is an instrument that measures and predicts changes in the quality of care and patients’ outcomes.

The main limitation of the study lies in the features of the validation process of the original tool with regards to the metric properties. Since in the original version of BERNCA instrument, the reliability and the validity of the questionnaire’s construct were provided but the reproducibility and sensibility to change were not evaluated. It should be pointed out as the strength of this study that it is the first one adapting the BERNCA-R into a Polish-language version and evaluating the reliability and validity of this tool for assessment of implicit rationing of nursing care among Polish nurses as well as our study is one of the few studies carried out in Europe. Further prospective studies, including direct assessment of causal relationships between implicit rationing of nursing care and patient outcomes, are still needed.

## Conclusions

The study using the BERNCA questionnaire demonstrated that the questionnaire may be used for investigating care rationing in groups of Polish nurses. Based on results of the Polish validation, considering cultural and regional differences, we can conclude that it is a reliable and valid tool that is recommended for use in Poland.

## Supporting information

S1 Fig Histogram of the total BERNCA-R score. (TIFF)

S1 File BERNCA-R database. (XLSX)
