# Supplementary figures and images for: Validation of Polish version of the Basel Extent of Rationing of Nursing Care revised questionnaire
Source: PLoS One. 2019 Mar 20;14(3):e0212918. doi: 10.1371/journal.pone.0212918 (PMC6426176; doi:10.1371/journal.pone.0212918)

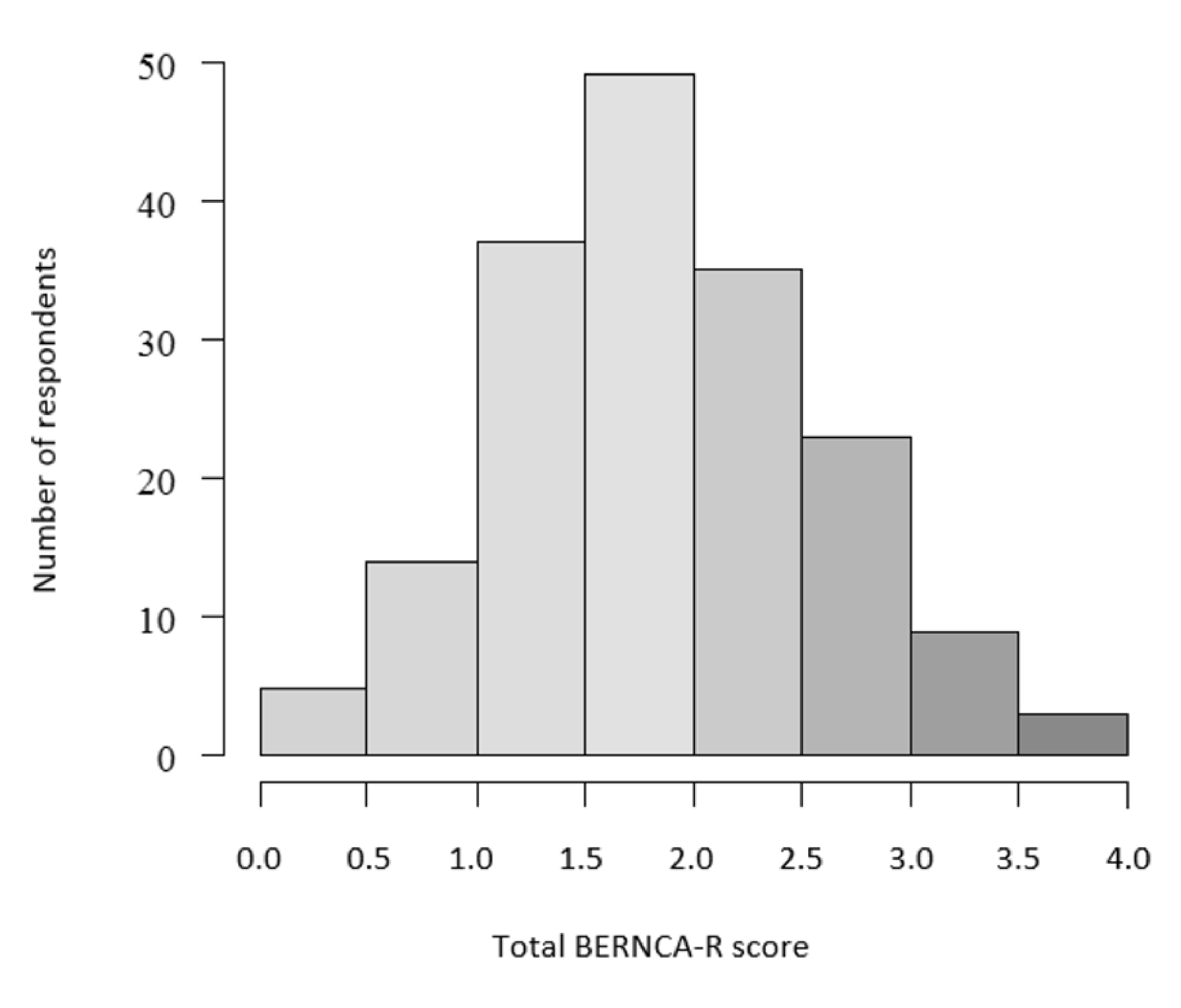

Supplement: S1 Fig — (TIFF) [file pone.0212918.s001.tiff]
